# Hexameric S100A12 is required for pro-inflammatory TLR4-signalling

**DOI:** 10.1186/1546-0096-13-S1-O30

**Published:** 2015-09-28

**Authors:** C Kessel, S Fühner, S Brockmeyer, H Wittkowski, D Föll

**Affiliations:** 1 University Childrens Hospital Münster, Pediatric Rheumatology & Immunology, Münster, Germany

## Introduction

The human granulocyte-specific Ca^2+^-binding protein S100A12 is particularily over-expressed in autoinflammatory diseases such as juvenile idiopathic arthritis (JIA) as well as other inflammatory conditions (i.e. infections, vasculitides) and has been ascribed to the group of pro-inflammatory damage associated molecular pattern molecules (DAMPs). In order to operate as DAMP, S100A12 requires binding to cellular receptors. Although the protein was originally found to bind the receptor of advanced glycation endproducts (RAGE), we recently demonstrated S100A12 to stimulate proinflammatory cytokine production in monocytes via TLR4 instead of RAGE.

## Objectives

DAMP:TLR4 signalling is often discussed controversial. Mechanistic insights into the protein: receptor interaction as available for HMGB1, for example, can help to explain the powerful pro-inflammatory potential of these proteins. A peculiarity of granulocytic S100A12 is it's oligomerization into di-, tetra- or hexamers upon Ca^2+^ and Zn^2+^-binding. In this study we assessed the mechanism of the S100A12:TLR4 interaction for these individual protein complexes.

## Methods

We performed extensive chemical crosslinking studies to assess S100A12 oligomerisation of both recombinant as well as native protein in autoinflammatory patients’ sera as well as protein directly isolated from granulocytic cytosol. For receptor-interaction studies, defined LPS-free chemically crosslinked S100A12-complexes were isolated via combined HPLC and gel filtration. TLR4-binding and signalling was tested on receptor-expressing cell lines as well as primary human cells. Cytokine expression in response to stimulation was quantified on mRNA and protein level.

## Results

In our assays, only combined presence of Ca^2+^ and Zn^2+^ concentrations in extracellular ionic-strength could induce S100A12 hexamer-formation. Ca^2+^/Zn^2+^-levels within physiological intracellular range could only induce oligomerisation up to the tetrameric complex. Correspondingly, we could detect hexameric S100A12 in patients’ serum, while we did not find this particular protein complex inside granulocytes. In vitro binding assays as well as cell stimulation experiments using chemically crosslinked HPLC-separated S100A12-oligomers revealed the S100A12-hexamer as the paramount TLR4-targeting pro- inflammatory active complex. Stimulation could be abrogated by interfering with TLR4-binding and, in particular, by blocking access to CD14.

## Conclusion

We have identified the S100A12-complex, which is responsible for pro-inflammatory signalling through TLR4. This is of great interest for designing improved diagnostics as well as precisely targeted therapeutic approaches, as currently tested with us.

